# AmpC β-Lactamases in SPICE (Serratia, Pseudomonas, Indole-Positive Proteus, Citrobacter, Enterobacter) Organisms: A Rising Threat to Antimicrobial Therapy

**DOI:** 10.7759/cureus.109750

**Published:** 2026-05-27

**Authors:** Prajakta S Jadhav, Priyanka M Mane, Satish Patil

**Affiliations:** 1 Department of Microbiology, Krishna Institute of Medical Science, Krishna Vishwa Vidyapeeth (Deemed to be University), Karad, IND

**Keywords:** ampc β-lactamase, antimicrobial resistance (amr), antimicrobial stewardship, enterobacterales, extended-spectrum β-lactamases (esbls), multidrug resistance (mdr), plasmid-mediated ampc, spice organisms

## Abstract

The global rise of antimicrobial resistance (AMR) among Gram-negative bacteria is a major public health threat. AmpC β-lactamases, particularly in SPICE (*Serratia, Pseudomonas, *indole-positive* Proteus, Citrobacter, Enterobacter*) organisms, contribute significantly to multidrug resistance by hydrolyzing cephamycins and other β-lactam antibiotics. Unlike extended-spectrum β-lactamases (ESBLs), AmpC β-lactamases are generally not inhibited by classical β-lactamase inhibitors such as clavulanic acid, sulbactam, and tazobactam. However, newer non-β-lactam β-lactamase inhibitors, including avibactam and relebactam, demonstrate activity against many AmpC-producing organisms, thereby complicating treatment strategies. This review highlights the molecular mechanisms, epidemiological patterns, clinical implications, detection methods, and emerging therapeutic options for AmpC β-lactamases in SPICE organisms. The review underscores the urgent need for enhanced surveillance, rapid diagnostics, and antimicrobial stewardship to manage infections caused by these resistant pathogens.

## Introduction and background

The growing threat of antimicrobial resistance (AMR) in Gram-negative bacteria has emerged as a critical global health crisis. The World Health Organization (WHO) has identified AMR as one of the top 10 global public health threats facing humanity, with multidrug-resistant (MDR) Gram-negative organisms contributing significantly to mortality, prolonged hospital stays, and increased healthcare costs [1]. Among the various mechanisms conferring resistance, β-lactamase production remains the most prevalent and clinically significant, leading to treatment failures with commonly used β-lactam antibiotics such as penicillins, cephalosporins, and carbapenems [2]. β-lactamases are classified according to the Ambler molecular classification (Table 1) system into four major classes.

**Table 1:** Ambler classification of β-lactamases and their major characteristics* ^*^[3-4] KPC: *Klebsiella pneumoniae* carbapenemase; EDTA: ethylenediaminetetraacetic acid

Ambler class	Enzyme type	Common examples	Major antibiotics hydrolyzed	Important characteristics
Class A	Extended-spectrum β-lactamases (ESBLs) and serine carbapenemases	TEM, SHV, CTX-M, KPC	Penicillins, cephalosporins, monobactams, and some carbapenems	Possess a serine residue at the active site; KPC enzymes are important carbapenemases associated with multidrug resistance
Class B	Metallo-β-lactamases (MBLs)	NDM, VIM, IMP	Broad-spectrum β-lactams, including carbapenems (except monobactams)	Require zinc ions for activity; inhibited by metal chelators such as EDTA but not by clavulanic acid
Class C	AmpC β-lactamases (cephalosporinases)	CMY, DHA, FOX, ACT, MIR	Cephamycins, penicillins, broad-spectrum cephalosporins	Usually chromosomally encoded or plasmid-mediated; poorly inhibited by β-lactamase inhibitors like clavulanic acid
Class D	Oxacillinases (OXA-type β-lactamases)	OXA-23, OXA-48, OXA-58	Oxacillin, penicillins, and some carbapenems	Some OXA-type enzymes exhibit carbapenem-hydrolyzing activity and are increasingly reported in multidrug-resistant Gram-negative bacteria

AmpC β-lactamases, first identified in the 1940s in *Escherichia coli (E. coli)* as enzymes capable of hydrolyzing penicillin, are classified under Ambler class C and Bush-Jacoby Group 1, and are of particular concern due to their ability to hydrolyze cephamycins (e.g., cefoxitin), penicillins, and extended-spectrum cephalosporins (e.g., cefotaxime, ceftazidime), while remaining resistant to inhibition by β-lactamase inhibitors like clavulanic acid, sulbactam, and tazobactam [5]. AmpC β-lactamase production can occur through three distinct mechanisms: chromosomally encoded inducible AmpC genes, found in organisms such as *Enterobacter cloacae, Serratia marcescens, Citrobacter freundii*, and *Pseudomonas aeruginosa;* chromosomal AmpC overexpression may result from mutations in regulatory regions, including promoter/attenuator alterations and upstream insertion sequence-mediated activation; or attenuator regions, typically seen in species like *Escherichia coli* and *Acinetobacter baumannii*. Plasmid-mediated AmpC (pAmpC) enzymes, which contribute to resistance in organisms such as *Klebsiella pneumoniae *and various *Salmonella* species [2].

Exposure to β-lactams can trigger a cascade of events leading to significant AmpC production and β-lactam resistance, even for infections caused by initially susceptible isolates. The risk of inducing AmpC production varies by β-lactam and by species, complicating treatment decisions. Importantly, pAmpC enzymes such as CMY, FOX, DHA, and ACT are now widely distributed among *Enterobacterales*, often coexisting with other resistance determinants [6-7].

A group of bacteria collectively referred to as SPICE (*Serratia spp., Pseudomonas aeruginosa, *indole-positive* Proteus spp*. (notably *Proteus vulgaris*), *Citrobacter spp.,* and *Enterobacter spp) organisms *are well known for harboring inducible chromosomal AmpC genes. The likelihood of clinically significant AmpC derepression varies among organisms traditionally grouped under the SPICE acronym, with species such as *Enterobacter cloacae* complex demonstrating higher clinical relevance compared with some other members, resulting in therapeutic failure even when initial susceptibility testing appears favourable [8]. SPICE organisms are frequently implicated in nosocomial infections, including bloodstream infections, catheter-associated urinary tract infections, ventilator-associated pneumonia, and wound infections, particularly among immunocompromised patients and those in ICUs [9].

Despite the clinical significance of AmpC-producing SPICE organisms, their detection remains a major diagnostic challenge. Routine susceptibility tests often fail to differentiate AmpC from ESBLs or report false susceptibility to third-generation cephalosporins, especially in the absence of a standardized confirmatory method [10]. Moreover, insufficient awareness and inadequate surveillance of AmpC β-lactamase mechanisms have facilitated their unnoticed spread within hospital environments, leading to limited therapeutic options and increased dependence on reserve antibiotics such as carbapenems.

This review aims to comprehensively evaluate the epidemiology, molecular mechanisms, diagnostic approaches, and therapeutic implications of AmpC β-lactamases in SPICE organisms. Given their increasing prevalence, diagnostic complexity, and clinical consequences, a focused analysis is necessary to enhance understanding, guide appropriate antimicrobial use, and inform policy and infection control measures.

## Review

Search strategy

A literature search was conducted using PubMed, Scopus, Google Scholar, and Web of Science databases for studies published up to December 2025. Keywords used included ‘AmpC β-lactamase’, ‘SPICE organisms’, ‘Enterobacterales’, ‘AmpC resistance’, and ‘plasmid-mediated AmpC’. English-language articles relevant to epidemiology, molecular mechanisms, detection methods, and therapeutic management were included. Duplicate publications, conference abstracts without full text, and studies lacking direct relevance were excluded. Approximately 180 articles were initially screened, of which 63 relevant articles, reviews, and clinical guidelines were included in the final review.

Epidemiology

The global rise of AmpC β-lactamase-producing organisms, particularly among SPICE group bacteria, poses a serious challenge to antimicrobial therapy. The prevalence of AmpC β-lactamases has increased significantly over the past two decades, driven by the selective pressure of broad-spectrum cephalosporins and the spread of pAmpC genes.

Global epidemiological trends

Chromosomal AmpC production has long been recognized as an intrinsic resistance mechanism in SPICE organisms. For example, studies report that 30-80% of *Enterobacter spp*., particularly *Enterobacter cloacae*, express inducible or derepressed chromosomal AmpC enzymes, depending on the degree of antimicrobial exposure [5,11]. Similarly, *Citrobacter freundii, Serratia marcescens*, and *Pseudomonas aeruginosa* possess inducible chromosomal AmpC genes that can become hyperexpressed via regulatory mutations [8].

In contrast, plasmid-mediated AmpC genes - such as bla<sub>CMY</sub>, bla<sub>DHA</sub>, bla<sub>FOX</sub>, and bla<sub>ACC</sub> - have been increasingly identified in* Escherichia coli, Klebsiella pneumoniae*, and SPICE organisms globally. These pAmpC genes originated from chromosomal AmpC genes of organisms like *Citrobacter freundii* and were mobilized onto plasmids, facilitating rapid horizontal gene transfer across species [6,12].

A multinational survey (SMART study) found that 7-10% of Enterobacterales isolates from hospitalized patients harbored pAmpC genes, with CMY-2 being the most frequently detected variant in North America, Europe, and Asia-Pacific regions [13]. In Southeast Asia and India, reports suggest even higher prevalence rates - CMY and DHA types being dominant - with multiple studies linking these genes to mobile elements like integrons and transposons [14-15].

Indian Epidemiological Data

India is among the countries with high burdens of antimicrobial resistance, and AmpC-producing SPICE organisms are frequently isolated from ICU settings, catheter-associated urinary tract infections, bloodstream infections, and surgical site infections [16]. A study from AIIMS, New Delhi, reported AmpC β-lactamase production in 38.2% of *Enterobacter spp*. and 29.4% of *Citrobacter spp*. isolates [17]. Another multicentre study from southern India demonstrated a 27-40% prevalence of AmpC enzymes in nosocomial Gram-negative isolates, with significant co-production of extended-spectrum β-lactamases (ESBLs), complicating phenotypic detection and treatment [18]. Plasmid-mediated AmpC-producing organisms have also been increasingly reported beyond hospital-associated settings. This shift reflects antibiotic misuse in both hospital and outpatient settings and underlines the need for national surveillance programs targeting AmpC resistance specifically.

Molecular mechanisms of AmpC β-lactamase-mediated resistance

Classification and Biochemical Properties

**Figure 1:**
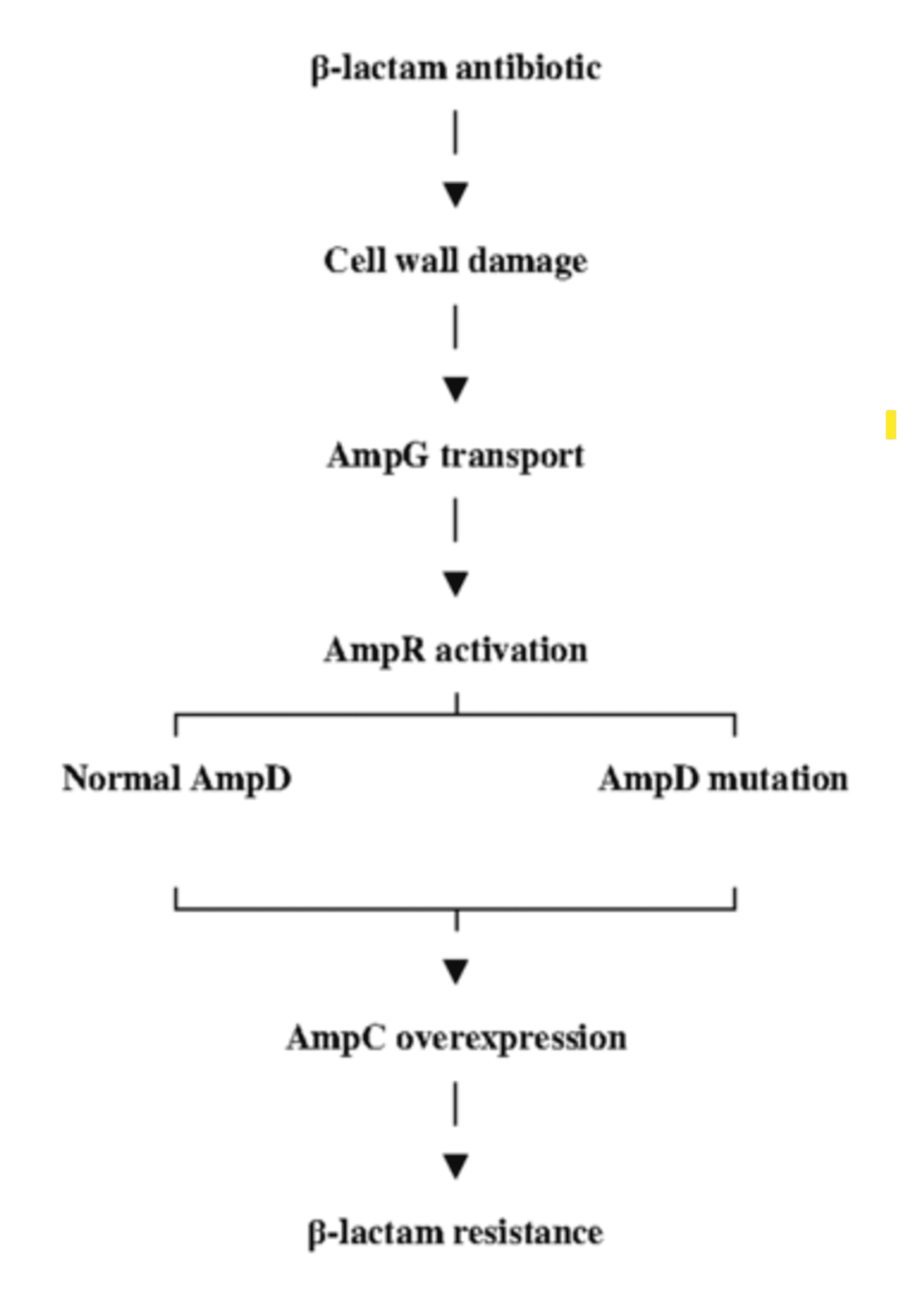
Simplified regulatory mechanism of inducible AmpC β-lactamase expression Image credit: authors

AmpC β-lactamases are Ambler class C enzymes that hydrolyze β-lactam antibiotics, particularly cephamycins and extended-spectrum cephalosporins, and are not inhibited by classical β-lactamase inhibitors such as clavulanic acid, sulbactam, or tazobactam [5]. These enzymes are either chromosomally encoded or plasmid-mediated, and their expression is regulated by complex molecular pathways. The simplified regulatory mechanism of inducible AmpC β-lactamase expression is presented in Figure 1.

Chromosomally Mediated AmpC Resistance

Several members of the SPICE group - *Enterobacter cloacae*, *Citrobacter freundii*, *Serratia marcescens*, and *Pseudomonas aeruginosa *- harbor intrinsic ampC genes located on the chromosome. These genes are typically under inducible regulation by the AmpR-AmpD-AmpG system [2]. In these organisms, the AmpC gene is typically located downstream of a regulatory region containing ampR (a LysR-type transcriptional regulator) and AmpG (a permease involved in recycling of cell wall fragments) [19]. Induction occurs when exposure to certain β-lactams (e.g., cefoxitin, imipenem) leads to cell wall degradation, generating muropeptide fragments that are transported into the cytoplasm by AmpG. These fragments interact with AmpR, converting it from a transcriptional repressor to an activator, leading to AmpC overexpression [20]. Derepressed mutants arise from mutations in AmpD or the AmpC promoter/attenuator region, resulting in constitutive AmpC expression and high-level resistance, even in the absence of inducers [21].

Plasmid-Mediated AmpC Resistance

pAmpC likely originated from chromosomal AmpC genes of *Citrobacter freundii​​, Enterobacter cloacae*, and other Enterobacterales, later mobilized by insertion sequences and transposons [6]. Common plasmid-encoded families include bla<sub>CMY</sub>, bla<sub>DHA</sub>,bla<sub>FOX</sub>, and bla<sub>ACC</sub> [7]. These genes are often associated with conjugative plasmids, enabling horizontal gene transfer between species such as *Klebsiella pneumoniae*, *Salmonella enterica*, and *Escherichia coli* [22]. pAmpC frequently coexist with ESBL genes (e.g., bla<sub>CTX-M</sub>) and carbapenemases (e.g., bla<sub>NDM</sub>), compounding multidrug resistance [23].

Isolate-Specific Mechanisms in SPICE Organisms

*Serratia marcescens*: This organism possesses intrinsic resistance to polymyxins mediated primarily through lipopolysaccharide (LPS) modification mechanisms, while resistance to first- and second-generation cephalosporins is associated with chromosomal AmpC β-lactamase production. It exhibits chromosomal inducible AmpC expression, occasionally supplemented by plasmid-mediated genes, characterized by low basal activity that can be rapidly induced in the presence of β-lactam antibiotics [24].

*Pseudomonas aeruginosa: *It carries a chromosomal AmpC gene (bla<sub>PDC</sub>) that is normally expressed at low basal levels but becomes upregulated when exposed to β-lactam antibiotics. The chromosomal AmpC expression is controlled by AmpR, and mutations in AmpD result in its overproduction, leading to resistance against cephalosporins and aztreonam [25].

Indole-positive* Proteus spp. (Proteus vulgaris​​​, Morganella morganii, Providencia spp.)*: These inherently possess chromosomal AmpC genes with inducible expression; however, cases of clinical isolates exhibiting AmpC hyperproduction are relatively uncommon [26].

*Citrobacter freundii: *It* *possesses a strongly inducible chromosomal AmpC gene and serves as a major source for the emergence of numerous plasmid-mediated AmpC variants [27]. 

*Enterobacter cloacae *complex*: *It* o*ften develops derepressed mutants, making it a frequent contributor to cephalosporin treatment failures [28].

Co-resistance Mechanisms

SPICE organisms often possess several resistance mechanisms in addition to AmpC β-lactamases, resulting in complex multidrug-resistant profiles. Common co-resistance strategies include: 

Porin modifications*:* They decrease antibiotic uptake and increase resistance levels.

Efflux pump overexpression*:* This confers resistance to multiple antibiotic classes simultaneously.

Alterations in target sites*:* mutations in DNA gyrase and topoisomerase enzymes.

Production of additional β-lactamases: including ESBLs and carbapenemases.

Phenotypic and genotypic detection methods for AmpC β-lactamases

Accurate identification of AmpC β-lactamase production in SPICE organisms is essential for guiding effective therapy and monitoring resistance trends. However, distinguishing AmpC producers from those producing ESBLs or carbapenemases remains difficult due to the frequent coexistence of these resistance mechanisms and the absence of Clinical and Laboratory Standards Institute (CLSI) or European Committee on Antimicrobial Susceptibility Testing (EUCAST)-approved confirmatory tests for AmpC detection [5]. Consequently, a combined approach using both phenotypic screening and molecular methods is generally advised [29]. The advantages and limitations of commonly used phenotypic methods for AmpC detection are shown in Table 2.

**Table 2:** Phenotypic methods for AmpC detection* ^*^[30-33,36] ESBL: extended-spectrum β-lactamase

Method	Principle	Advantages	Limitations
Cefoxitin screening	Reduced susceptibility	Simple, cheap	Low specificity
AmpC disc test	Inhibition by cloxacillin/boronic acid	Detects plasmid AmpC	May miss inducible chromosomal AmpC
Three-dimensional test	Distortion of the inhibition zone	Useful for hyper producers	Labor-intensive, not standardized
Inhibitor-based assays	Boronic acid vs. cefoxitin synergy	Differentiates ESBL+AmpC	Not widely standardized

Phenotypic Detection Methods

Cefoxitin screening test: The test organism is swabbed on Mueller-Hinton agar (MHA), a cefoxitin disc is placed on it, and incubated at 37°C for 16-18 hours. Reduced Susceptibility to the cefoxitin test is used as a screening test. If the zone size of cefoxitin was <18 mm, then the isolate was further tested for the production of AmpC [30].

AmpC disk test: A standard *Escherichia coli* strain ATCC (25922) is taken, and a 0.5 McFarland is made. This is inoculated on MHA. Then a sterile disc is soaked in 20 µL saline, and a few colonies of the test organisms are inoculated. This disc is then placed next to the cefoxitin disc, nearly touching the disc on the inoculated plate. This plate is kept for overnight incubation. If there is a flattening zone around the disc or an indentation around cefoxitin, then the organism is considered an AmpC producer [31].

Disk approximation test (induction-based method): Prepare a bacterial suspension equivalent to 0.5 McFarland standard and evenly inoculate the surface of an MHA plate using a sterile cotton swab. Place a 30 µg ceftazidime disc at the centre of the plate, then 10 µg imipenem, 30 µg cefoxitin, and 20/10 µg amoxicillin/clavulanate disc were placed at a distance of 20 mm from the ceftazidime disc. Invert the inoculated plate and incubate at 37 °C for 18-24 hours. After incubation, examine the zone of inhibition. Blunting or flattening of the ceftazidime inhibition zone toward any of the adjacent discs indicates AmpC β-lactamase induction, signifying a positive result [32].

AmpC tris EDTA disc test: This method utilizes Tris-EDTA to permeabilize bacterial cells, allowing the release of β-lactamase enzymes into the surrounding medium. AmpC discs are prepared by adding 20 µL of a 1:1 mixture of saline and Tris-EDTA onto sterile filter paper discs, which are then dried and stored under refrigeration. Before testing, the discs are rehydrated with 20 µL of saline, and a few colonies of the test organism are applied to each disc. A 30 µg cefoxitin disc is placed on a MHA plate previously inoculated with *Escherichia coli* ATCC strain. The prepared AmpC disc is positioned in proximity to the cefoxitin disc, with the inoculated surface facing the agar. The plate is inverted and incubated at 35 °C for 18-24 hours. Following incubation, the presence of indentation or flattening of the cefoxitin inhibition zone near the AmpC disk signifies enzymatic inactivation of cefoxitin and is interpreted as a positive result for AmpC β-lactamase production [33].

Modified three-dimensional tests: In a micro centrifuge tube with peptone water, a suspension of fresh overnight growth is made and is pelleted by centrifuging at an rpm of 3000 for 15 mins. Then this suspension is freeze-thawed seven times to yield the enzyme. ATCC (25922) of *Escherichia coli*strain is taken, and a 0.5 McFarland is made and inoculated on MHA. A Cefoxitin disk of (30 µg) is placed, and a straight slit of 3cm is made with a sterile blade 3 mm from the edge of the disk. On the other side of the slit, wells are made and loaded with enzyme extract of 30-40 µL, and the plate is kept in a straight position for 5-10 mins until the solution dries. Then the plates are placed for overnight incubation at 37ºC. Isolates showing a distorted zone of inhibition around the cefoxitin disk are considered as AmpC producers [34].

Inhibitor-based methods

Cefoxitin-Cloxacillin Double Disc Synergy Test

The CC-DDS is performed to detect AmpC β-lactamase production based on the inhibitory action of cloxacillin on AmpC enzymes. An MHA plate is inoculated with a 0.5 McFarland standard bacterial suspension. Two discs are placed on the agar surface, one containing cefoxitin (30 µg) alone and another containing cefoxitin (30 µg) supplemented with 200 µg (20 mg/mL) of cloxacillin. The plate is then incubated at 37 °C for 18-24 hours. After incubation, an increase of ≥4 mm in the inhibition zone diameter around the cefoxitin-cloxacillin disk compared to the cefoxitin-only disc indicates AmpC β-lactamase production [35].

Boronic Acid Disc Test

The test organism is incubated overnight, and a 0.5 McFarland is made. It is inoculated on MHA. Two discs of 30 µg of cefoxitin are placed on the agar plate. On one of the discs, add 20 µL of phenylboronic acid and let it absorb. Keep the plate for incubation at 35℃ overnight. If an increase of (>= 5mm) zone diameter around the disc with phenylboronic acid is noted, then the organism is considered an AmpC producer [36].

Genotypic detection methods

Genotypic methods serve as the gold standard for detecting AmpC β-lactamase genes and differentiating chromosomal (intrinsic) AmpC from plasmid-mediated (pAmpC) variants in SPICE organisms (*Serratia, Pseudomonas, indole-positive Proteus/Providencia/Morganella, Citrobacter,* and *Enterobacter*). These molecular techniques overcome the limitations of phenotypic tests, allow precise identification of AmpC families such as blaCMY, blaDHA, blaFOX, blaACC, blaMOX, and blaEBC/ACT/MIR, and help clarify co-existing resistance mechanisms as well as trace epidemiological patterns. However, their accuracy depends on the use of well-designed primers, reliable sequence databases, and cautious interpretation to differentiate between gene presence and actual expression [5,9].

Multiplex PCR

Detection of plasmid-mediated AmpC β-lactamases is performed using the gold-standard multiplex PCR assay, which simultaneously targets the six major AmpC gene families through six specific primer sets [7]. The amplification protocol begins with an initial denaturation at 94°C for 3 minutes, followed by 25 cycles consisting of denaturation at 94°C for 30 seconds, annealing at 64°C for 30 seconds, and extension at 72°C for 1 minute. A final extension step at 72°C for 7 minutes completes the reaction. A 100-1000 bp DNA ladder serves as the molecular size marker. Primer details are listed in Table 1. Amplified products are visualized by 2% agarose gel electrophoresis [37]. In *Pseudomonas aeruginosa*, resistance is generally attributed to chromosomal AmpC (PDC) variants rather than plasmid-mediated AmpC; several Indian clinical reports have confirmed PDC-type AmpC activity in this species [38]. Globally, and also in India, the most frequently encountered plasmid-mediated AmpC families are CMY (particularly CMY-2 and CMY-42) and DHA-1, both of which have been identified in isolates from clinical and environmental sources [38]. Representative primers employed for PCR-based genotypic identification of AmpC β-lactamase families are shown in Table 3.

**Table 3:** Representative primers for genotypic detection of AmpC β-lactamases* ^*^[7]

Family	Representative genes	Forward primer (5′–3′)	Reverse primer (5′–3′)	Amplicon size (bp)	Reference
CIT family	bla_CMY-2_, bla_LAT_	TGG CCA GAA CTG ACA GGC AAA	TTT CTC CTG AAC GTG GCT GGC	462 bp	Pérez-Pérez and Hanson, 2002
DHA family	bla_DHA-1_, bla_DHA-2_	AAC TTT CAC AGG TGT GCT GGG T	CCG TAC GCA TAC TGG CTT TGC	405 bp	Pérez-Pérez and Hanson, 2002
FOX family	bla_FOX-1_ to bla_FOX-5_	AAC ATG GGG TAT CAG GGA GAT G	CAA AGC GCG TAA CCG GAT TGG	190 bp	Pérez-Pérez and Hanson, 2002
ACC family	bla_ACC-1_	AAC AGC CTC AGC AGC CGG TTA	TTC GCC GCA ATC ATC CCT AGC	346 bp	Pérez-Pérez and Hanson, 2002
EBC family	bla_ACT_, bla_MIR_	TCG GTA AAG CCG ATG TTG CGG	CTT CCA CTG CGG CTG CCA GTT	302 bp	Pérez-Pérez and Hanson, 2002
MOX family	blaMOX-1, blaMOX-2	GCA ACA ACG ACA GGA AAC AGG C	CAA CTC TTC GCC GCA TGA C	520 bp	Pérez-Pérez and Hanson, 2002

Whole-Genome Sequencing

Whole-genome sequencing (WGS) provides the most comprehensive high-resolution view of the genetic determinants that underpin antimicrobial resistance, virulence, and the epidemiology of bacterial pathogens. In the context of SPICE organisms (*Serratia, Pseudomonas, *indole-positive* Proteae, Citrobacter, Enterobacter*), WGS is uniquely valuable because these taxa combine chromosomal resistance mechanisms (inducible ampC, regulatory mutations) with an increasing burden of plasmid-mediated resistance determinants (pAmpC, ESBLs, carbapenemases) [7,27]. WGS enables simultaneous detection and genomic localization of ampC genes and their mobile genetic contexts, precise identification of promoter/regulatory mutations that drive derepression, plasmid replicon and transposon mapping, strain typing, and high-resolution phylogenetics for outbreak and transmission investigations [7].

Although conventional multiplex PCR assays remain widely used for the detection of plasmid-mediated AmpC genes, recent advances in molecular diagnostics increasingly utilize WGS-based tools. Short-read sequencing platforms such as Illumina provide highly accurate resistance gene identification, whereas long-read technologies, including Oxford Nanopore and PacBio, facilitate plasmid reconstruction and structural analysis of resistance determinants and bioinformatic platforms such as ResFinder, CARD (Comprehensive Antibiotic Resistance Database), and NCBI AMRFinderPlus for rapid identification and characterization of antimicrobial resistance determinants.

Clinical implications and therapeutic challenges

The emergence of AmpC β-lactamase-producing SPICE organisms has profound implications for clinical practice, infection control, and antimicrobial stewardship. Infections caused by these organisms are often associated with increased morbidity, mortality, prolonged hospital stay, and higher healthcare costs [39-40].

Therapeutic Limitations

The therapeutic landscape for AmpC-producing SPICE organisms remains severely constrained. These enzymes confer resistance to penicillins, first- through third-generation cephalosporins, and aztreonam, while maintaining variable activity against fourth-generation cephalosporins [41,5]. Co-resistance mechanisms, including porin alterations, efflux pump upregulation, and co-production of other β-lactamases, further limit treatment options [42]. Recent Infectious Diseases Society of America (IDSA) guidance recommends organism-specific treatment decisions for AmpC-producing Enterobacterales and supports the use of cefepime as a carbapenem-sparing option in selected clinically stable isolates with confirmed susceptibility [43]. Carbapenems have traditionally served as the primary therapeutic option for serious AmpC-mediated infections. However, the increased utilization of Carbapenems has contributed to the emergence of carbapenem-resistant Enterobacteriaceae (CRE), creating a concerning cycle of resistance development [44]. Non-β-lactam alternatives, including fluoroquinolones and aminoglycosides, are frequently compromised by co-resistance mechanisms, leaving clinicians with limited oral options for step-down therapy [45].

The selection of appropriate empirical antimicrobial therapy for suspected SPICE organism infections requires careful consideration of patient risk factors, institutional epidemiology, and local resistance patterns [46]. Traditional empirical regimens utilizing third-generation cephalosporins are inadequate for patients with risk factors for AmpC-producing pathogens, including prolonged hospitalization, prior antibiotic exposure, and presence of medical devices [47].

Novel Therapeutic Approaches

The escalating clinical importance of AmpC β-lactamases in SPICE organisms has stimulated the development of multiple new strategies that either (a) restore activity of existing β-lactams by inhibition of the enzyme, (b) use novel β-lactams that bypass common resistance mechanisms, or (c) employ non-traditional antimicrobials and gene-targeting approaches. 

Next-generation β-lactamase inhibitors and β-lactam/β-lactamase inhibitor (BL/BLI) combinations: New BL/BLI combinations have been the most immediately translatable option for treating AmpC-producing pathogens. Novel β-lactamase inhibitors such as avibactam and relebactam demonstrate inhibitory activity against Class C (AmpC) β-lactamases. In contrast, vaborbactam primarily exhibits activity against Class A KPC carbapenemases and has limited clinical activity against AmpC-producing organisms. These agents differ in spectrum and clinical niche (for example, ceftazidime-avibactam has broad utility; imipenem-relebactam and meropenem-vaborbactam have specific roles) and should be selected according to organism, resistance mechanisms, and local susceptibility [48-49]. Bicyclic boronate inhibitors represent a notable advance: taniborbactam (VNRX-5133) is a broad-spectrum bicyclic boronate inhibitor able to inhibit many class A, C, and some class B/D enzymes in vitro and to restore activity of cefepime/meropenem in preclinical studies; human pharmacokinetic and early safety data are available, and clinical development continues. Taniborbactam-containing combinations therefore hold promise against AmpC and even against some carbapenemases in mixed resistance backgrounds [50-51].

Novel β-lactams and siderophore cephalosporins: Cefiderocol, a siderophore cephalosporin that uses iron-uptake pathways to penetrate Gram-negative bacteria, shows potent in vitro activity against a broad range of Gram-negative pathogens, including many AmpC producers, and has become an important option for difficult-to-treat infections. However, emerging reports of heterogeneous and geographic resistance highlight the need for stewardship and susceptibility testing before use [52-53].

Optimization of existing β-lactam therapy (carbapenem-sparing strategies): Clinical and observational data indicate that cefepime administered at higher dosing regimens (e.g., 2 g every 8 hours with prolonged infusion) or selected β-lactam/β-lactamase inhibitor combinations can serve as carbapenem-sparing options for selected AmpC bloodstream infections after careful phenotypic evaluation to exclude ESBL co-producers and in clinically stable patients. Cefepime (particularly when given at optimized dosing for severe infections) and some cefepime-based combinations (for example, cefepime + newer inhibitors) have been supported by comparative outcome studies and meta-analyses as reasonable options in specific settings, reducing carbapenem exposure while preserving efficacy. Similarly, cefepime-tazobactam and other tailored combinations are being explored as carbapenem-sparing therapies in vitro and in small clinical series. Clinical choice must be individualized and guided by MICs, site of infection, pharmacodynamics, and local ecology [54-56].

Discussion

AmpC β-lactamases have emerged as one of the most challenging resistance mechanisms among the SPICE group. Their ability to hydrolyze penicillins, cephalosporins, and monobactams while resisting inhibition by clavulanic acid has contributed to widespread treatment failures and multidrug resistance. The chromosomal location of AmpC genes in most SPICE members allows for inducible and derepressed expression, further complicating empirical therapy.

Epidemiological studies have shown that AmpC-producing isolates are increasingly prevalent in healthcare-associated infections, particularly in intensive care units and postoperative wards [57-58]. Co-production with extended-spectrum β-lactamases (ESBLs) or carbapenemases has further aggravated resistance trends, leading to reduced susceptibility even to fourth-generation cephalosporins and Carbapenems [59]. In India and other developing regions, the inappropriate use of broad-spectrum antibiotics and lack of molecular surveillance have accelerated the selection of plasmid-mediated AmpC enzymes, such as CMY, DHA, and FOX types [60,9]. Phenotypic detection of AmpC remains challenging, as routine screening methods may not differentiate between AmpC and ESBL producers. Advanced diagnostic approaches, such as multiplex PCR, microarray analysis, and WGS, are increasingly used to confirm genotypic variants and trace epidemiological links [60-61].

Therapeutic management is equally complex. Although Carbapenems have traditionally been the mainstay of treatment, their overuse has resulted in the emergence of carbapenemase-producing strains. Non-carbapenem alternatives such as cefepime, piperacillin-tazobactam, and ceftolozane-tazobactam have demonstrated partial success, particularly in isolates with low-level AmpC expression [8,9]. The urgent need for novel antimicrobials and inhibitor molecules underscores the critical role of continuous research and global collaboration. Effective infection control measures - such as antibiotic stewardship, strict hand hygiene, and regular surveillance - remain the backbone of containment strategies. Furthermore, understanding the genetic plasticity and regulatory mechanisms of AmpC expression is essential for developing targeted inhibitors and predictive diagnostic assays [61].

## Conclusions

AmpC β-lactamase-producing SPICE organisms have emerged as significant nosocomial pathogens, contributing to multidrug resistance and therapeutic failures. Their ability to hydrolyze a broad range of β-lactam antibiotics, coupled with frequent co-production of ESBLs, poses serious diagnostic and therapeutic challenges. Accurate detection through standardized phenotypic and molecular methods is vital for effective infection control and epidemiological surveillance. Strengthening antimicrobial stewardship, enforcing rational antibiotic policies, and implementing continuous surveillance are essential to curb their dissemination. A coordinated global effort among clinicians, microbiologists, and public health authorities is imperative to mitigate the escalating threat of AmpC-mediated resistance and safeguard the efficacy of current antimicrobial therapies.
